# Southern Surgical and Gynecological Association

**Published:** 1892-10

**Authors:** Thos. F. Wood


The meeting of the Southern Surgical and Gynecological Association, in Louisville, has been postponed from the 8th, 9th and 10th until the 15th, 16th and 17th of November. It was thought wise to change the time of the meeting from the fact that the 8th of November is the date of the Presidential election. Everything points to a very successful session.

